# “PrEP”ing for a PrEP demonstration project: understanding PrEP knowledge and attitudes among cisgender women

**DOI:** 10.1186/s12905-021-01348-8

**Published:** 2021-05-25

**Authors:** Elizabeth C. Pasipanodya, Jamila Stockman, Thupten Phuntsog, Sheldon Morris, Christina Psaros, Raphael Landovitz, K. Rivet Amico, David J. Moore, Jill Blumenthal

**Affiliations:** 1 Santa Clara Valley Medical Center, Santa Clara, CA USA; 2 University of California San Diego, 220 Dickinson Street Suite A, San Diego, CA USA; 3 Department of Psychiatry, Massachusetts General Hospital, Boston, MA USA; 4 UCLA Center for Clinical AIDS Research and Education, University of California Los Angeles, Los Angeles, CA USA; 5 Department of Health Behavior and Health Education, School of Public Health, University of Michigan, Ann Arbor, MI USA

**Keywords:** Pre-exposure prophylaxis (PrEP), Cisgender women, HIV prevention, PrEP attitudes

## Abstract

**Background:**

Prior to implementing a pre-exposure prophylaxis (PrEP) demonstration study, we sought to explore cisgender women’s experiences with HIV prevention, PrEP knowledge and attitudes, and anticipated barriers and facilitators for PrEP uptake and adherence in Southern California.

**Methods:**

Three focus groups were held with cisgender women of mixed HIV serostatus in San Diego and Los Angeles between November 2015 and January 2016. Women were recruited through local testing sites, community-based organizations, and social media. Focus groups were audio-recorded and transcripts were analyzed using thematic analysis.

**Results:**

Twenty-two women participated in focus groups, with median age 44 (IQR 30–53) and 6 identifying as non-Hispanic Black, 7 non-Hispanic White, 8 Latina and 1 mixed race. Despite limited prior PrEP knowledge and no PrEP experience, participants expressed interest in taking PrEP. Anticipated benefits were freedom from worry about HIV and control over sexual health; however, these were tempered by concerns including the possibility of increased HIV risk behaviors and potential side effects. Cisgender women reported potential barriers to PrEP uptake and adherence barriers, like competing priorities and poor PrEP access. Conversely, PrEP facilitators included utilizing practical tools such as phone apps and pill boxes as well as receiving encouragement from loved ones and support from other cisgender women on PrEP, women living with HIV and their medical providers.

**Conclusions:**

Although PrEP awareness was low, participants recognized the importance of PrEP and ways to facilitate adherence. Exploring perspectives of cisgender women is integral to developing effective interventions to support PrEP uptake and adherence for women at elevated risk for HIV.

**Supplementary Information:**

The online version contains supplementary material available at 10.1186/s12905-021-01348-8.

## Background

Combination tenofovir disoproxil fumarate with emtricitabine (TDF/FTC) is effective for HIV pre-exposure prophylaxis (PrEP). Since its approval in 2012, PrEP use among cisgender men has grown substantially [1]; however, cisgender women continue to have low PrEP uptake [2]. In 2017, cisgender women constituted 19% of the new HIV diagnoses in the US [3]. In addition, the majority of new diagnoses are among Black and Latina women [4, 5]. Preventing HIV among women, particularly among women of color, is essential to ending the HIV epidemic.

Unlike men who have sex with men (MSM) and transgender women (TGW), risk factors for cisgender woman's likelihood of acquiring HIV are less clearly defined and may differ from one individual to another. Living in high HIV prevalence areas, having a history of sexually transmitted infections (STIs), participating in transactional sex, experiencing intimate partner violence, and engaging in injection drug use (IDU) may all contribute to a woman’s lifetime risk for HIV acquisition [6]. Moreover, women may be unaware of their male sex partner’s risk factors, resulting in an underutilization of safe sex practices [7]. Improved and multi-faceted HIV prevention efforts, that include PrEP use, are needed to address HIV acquisition among cisgender women.

Cisgender women have been found to have lower awareness of PrEP than MSM [8]. One US study investigating the acceptability and feasibility of PrEP among at-risk women reported that less than 10% of participants had previously heard about PrEP and none were aware of its indication for HIV-prevention among women; however, once study participants learned about PrEP, many expressed interest in taking PrEP themselves [9]. Low baseline rates of PrEP awareness among cisgender women potentially obscure even lower rates of awareness among cisgender women of color despite their greater generalized risk for HIV.

PrEP awareness is only an initial barrier to wider PrEP usage among women, as women also face a myriad of barriers in accessing and adhering to PrEP. Insufficient provider knowledge and support, difficulty recognizing their risk for HIV, PrEP cost, HIV/AIDS stigma, and limited resources for HIV prevention all contribute to a framework that make acquiring and adhering to PrEP difficult for women [10]. Other work examining barriers to PrEP uptake among women in the US has identified low social support and difficulty communicating with health care providers about sexual matters as potential barriers impeding women’s access to PrEP [11].

In preparation for an open-label PrEP demonstration project among HIV-negative cisgender women in California, the present qualitative study conducted focus groups to better understand cisgender women's knowledge and attitudes towards PrEP and to obtain their perspectives on ways of facilitating other cisgender women’s PrEP uptake and adherence.

## Methods

### Study participants and recruitment

Prior to the implementation of a California HIV/AIDS Research Program (CHRP)-funded (grant numbers EI11-SD-005B and EI11-LA-002B) PrEP single arm demonstration study among cisgender women, three focus groups, (two in San Diego and one in Los Angeles) were conducted between November 2015 and January 2016. The decision to conduct focus groups was based on the desire to explore experiences in a group context, allowing for ideas to be considered from multiple perspectives and inviting the possibility for new direction or insights to emerge through group discussions. Each focus group was comprised of 4 to 10 individuals, with a total of 22 participants. Participants were recruited as part of a convenience sample through local HIV testing sites, community-based organizations that support cisgender women and social media. Inclusion criteria for participation were identification as a cisgender woman, 18 years of age or older, English-speaking, and able to provide informed consent. Our intention was to allow for variability in views to capture a breadth of opinions and experiences. There was an interest in including Black and Latina cisgender women in the study, but all women regardless of race or ethnicity were included. Although focus groups focused on the views of representative prospective PrEP users (i.e., cisgender women without HIV), four women living HIV (WLHIV) participated in the focus groups in order to foster inclusivity and maintain good relations in this community-based participatory project. To ensure the privacy of WLHIV, the informed consent included a discussion about keeping HIV status confidential.

### Focus groups

All focus groups were conducted in English, were approximately 90 min in duration, and held at clinical research sites. They were led by two facilitators in San Diego (J.K. and D.J.M.) and two facilitators in Los Angeles (R.A. and C.P.), all of whom had extensive experience in conducting qualitative research and were investigators for the planned CHRP-funded PrEP study. Additional study staff was present for administrative purposes. A light meal was provided at the beginning of each of the focus groups and all participants were compensated $35 at the end of the discussion for their time. Prior to each focus group discussion, participants completed a short form collecting data on self-reported HIV status, age, and race/ethnicity, and a facilitator provided basic information about PrEP, including its administration, mechanism of action, possible side effects, and clinical efficacy. Using a semi-structured guide developed for this study, facilitators asked open-ended questions to elicit discussion about PrEP awareness and knowledge, PrEP candidacy, PrEP benefits and concerns, PrEP facilitators and barriers to uptake and adherence, and ways to increase PrEP awareness among women. For instance, in the discussion of PrEP candidates, participants were asked, “Who do you think might benefit from taking PrEP?” To obtain views on PrEP adherence, participants were asked, “What or who helps you take your medications?” and “What things could get in the way or affect a woman’s ability to take PrEP?” (see Additional file 1 for the complete Interview Guide).

### Qualitative analysis

All focus groups were audio-recorded and transcribed verbatim by T.P. without identifying information. Following transcription, an inductive thematic analysis approach was carried out to identify emerging themes. All transcripts were independently coded by researchers (J.B. and E.P.) using the qualitative analysis software MAXQDA v.12 and a coding dictionary consisting of mutually-exclusive codes and memos was constructed [12]. Initial interrater reliability of codes was low, primarily due to differences in the frequency of subtheme coding between raters, resulting in further code refinement and assignment with some subthemes merged into broader themes. Differences in coding were resolved through discussion between the two raters and re-rating content with new codes. Following further coding review, final agreement and interrater reliability was high (Cohen’s kappa > 0.9) [13]. The manuscript was organized in accordance with the COREQ guidelines [14].

### Ethics approval and consent to participate

The research protocol was performed in accordance with the Declaration of Helsinki and approved by the Institutional Review Boards at the University of California Los Angeles and the University of California San Diego. All focus group participants provided written informed consent prior to participation.

## Results

Of the 22 participants, 6 identified as non-Hispanic Black, 7 as non-Hispanic White, 8 as Latina, and 1 as mixed race. Participants were a median age of 44 years old (Interquartile Ratio 30–53). Four WLHIV participated in the first focus group 1; the other focus groups were entirely comprised of women who did not have HIV (see Table 1). Several broad themes emerged from discussions among participants across the three focus groups (see Table 2). In particular, themes related to participants’ (i) PrEP awareness and suggestions of improving PrEP awareness among other women, (ii) concerns and benefits of PrEP use as well as their perceptions of who might benefit the most from PrEP use, and (iii) perceptions of barriers and facilitators of PrEP initiation and adherence were identified.

**Table 1:** Participant demographics

	N = 22
Age, median (IQR)	44 (30–53)
*Race/ethnicity*	
Non-Hispanic white	7
Non-Hispanic black	6
Latina	8
Other/mixed	1
*HIV serostatus*	
Living with HIV	4
Living without HIV	18

**Table 2:** Focus group domains and emerging themes/subthemes

Domain	Themes	Exemplar quotes
PrEP awareness	*PrEP misconceptions*	
	Suppresses HIV infection	That is a concern. Is it just keeping it in advance? … then you’re like, stop taking PrEP, and then you get, you’re like, “I have HIV?”
	Results in tolerance or resistance to medications	“You’re basically building an immune system to it, so these people going out here thinking, “Well, I can just take it when I plan on having sex and I’m good.” But not knowing that, “Well, I’m now building a resistance to it because I’m not taking it as prescribed.”
	*Common PrEP questions*	
	Efficacy	“Yeah. Okay. So, say you start taking this PrEP and your partner has HIV and you don’t, and you been takin’ PrEP ‘cause you know he has HIV. Like he’s been open about it. And then you been takin’ PrEP and then suddenly you get pregnant by him, is the baby gonna have HIV or not because you was takin’ PrEP while you started…?”
	Accessibility	“Do you have to have a prescription or can you buy it over the counter?”
	Safety and side effects	“Is it safe for pregnant women to take it?”
	Dosing	“So, is it like birth control? You know how birth control, you have to take it at that set time.”
	*Facilitating PrEP awareness*	
	Greater HIV education and HIV stigma reduction	“I also think it’s important that, and this is so much easier said than done, but to make it more acceptable. For example, I’m thinkin’ of Susan G. Komen. Someone who gets breast cancer is completely innocent. You, you know, and look at how not, look at all the exposure that breast cancer how has, and on the support, and millions and millions and millions of dollars that they’ve raised for breast cancer, for women. Why is it not okay for somebody to say, “I’m HIV-positive, but I’m not an alien, I’m not weird, I’m not different.”
	Word of mouth endorsements	“… if I was to come to you and say, as somebody that’s HIV-positive for 23 years. If they have a pill that can help the prevention of passing the virus on, as a single female, would you be interested in taking this pill?”
	Education in prisons and schools	…” they don’t care. Something needs to make them care. Bring it in the schools. Bring it in the middle schools. The high schools. The colleges.”
	Public advertisements	“…they’re gonna be advertisements and commercials. And, every time I’m watchin’, I’m watchin’ Hulu—do you know how many drug commercials I see?”
	Social media	“…that’s what I’m sayin’, if they just type somethin’ out and put it on Facebook, Instagram, and just share it. Whether they say somethin’ negative or positive about it, people’s still gonna talk about it and it’s gonna get out there in the world.”
	Combine with reproductive and other health services	“I had to go to Planned Parenthood and get my birth control. But, this wasn’t…proposed to me as an option. So, I wasn’t aware that this was out there. …I think if more people knew, they might be more interested. Also, you know, if you’re getting birth control…we should also be informed, ‘Oh, by the way…”
PrEP uptake	*PrEP concerns*	
	Side effects	“Well, before they give it to you, they come with a, it comes with side effects. Every medication has side effects that’s probably bein’ passed by the FDA…. you enterin’ basically at your own risk.…”
	Risk compensation	But you’re gonna get a lot of people, too, that are gonna think, ‘Well, if I take this tonight, I’m good. I can go out and just get as buck wild as I want.”
	Diversion/misuse	“I feel like, and people are always going to abuse it, people are going to sell it, all my, you know, in college you could buy anything you want—I’m not that far removed from it. You can find anything you want.”
	Poor adherence	“I think a lot of people will do that. They’ll just stop taking, “I’m gonna take it every three days ‘cause it’s in my system.”
	No protection from other STIs	“…there’s not just that, there’s like gonorrhea, there’s other things.”
	*PrEP benefits*	
	Protection from HIV	“… the only reason to take PrEP is to make sure I don’t get HIV.”
	Autonomy	“You might trust that person so you guys stop usin’ condoms. I would still take it if I’m sexually active like that because that’s my health.”
	Peace of mind	“So to help your health, and my frame of mind that you’re gonna be semi-protected, that’s the situation I would use it in for myself.”
	More preventative choices	But it gives them like the option because I know when I was in high school they only taught abstinence, that’s it… I need to have, not everyone’s going to be on a straight and narrow path… And, everyone needs to have, you know, the option.”
	Facilitating intimate relationships	“Now, that opens the door for me further down the line if I choose to have a relationship, I can bring this to my partner that, “Hey, this is my situation. This is what we can do protect…”
	*PrEP candidates*	
	Women in serodiscordant relationships	“So because I don’t want you to catch anything, I would then ask for you to take the PrEP.”
	Women with multiple sex partners or in relationship with a non-monogamous partner	“I know when I was between the 18–24, that’s the most when you want to venture out, and… you want to get out of your parents’ like—not eye—but like you want to explore the world for yourself, so with that comes like risky behavior, and multiple partners, and just all different situations.”
	Women who use drugs or engage in sex work	there’s people that’s at high risk for AIDS, for HIV. If you’re prostitutin’, usin’ drugs, or whatever you’re doing, your lifestyle..”
	Women of color	Well, people of color, it’s supposed to be more for people of color
	Youth	“… teenagers and stuff, they’re more, is catchin’ it now and stuff, so like for them, I guess it would be, you know, something good.”
	Older women	You know where, I think older women, it would be awesome for, you know, for PrEP to be targeted at the older women that are not goin’ through menopause, that are not using any kind of, which is the next growing population…”
Barriers and facilitators of PrEP initiation and adherence	*Facilitators*	
	Maintaining health	“So, with that just bein’ said, it’s like, exactly, why not do somethin’ that’s, could prevent you from layin’ in a hospital. Like I said, ‘cause if I was to take it, there will be nothing that would distract me from taking it…”
	Sense of empowerment in directing one’s sexual health	Like I, like at the end of the day, it’s gonna fall on me, like regardless of what the doctor says, regardless of whatever happened between me getting the pill and me taking it. Like at the end of the day, it’s my responsibility.”
	Support from family/friends	“If I am open, like especially with people close to me about that I’m taking it, I think that would help me…keep taking it because then you go a support system behind you.”
	Support from medical providers	“I think if I trust my healthcare provider, I’m more likely to be honest about what the issues are for me to not take it, or what’s getting in the way, or what’s, or maybe that I am taking it correctly.”
	Support from women taking PrEP	“I just need some ideas, to bounce ideas, to be comfortable to talk to somebody who takes the pill. ‘Cause, you’re not gonna have that same conversation with someone who don’t even know nothin’ about PrEP, don’t even take PrEP.”
	Hearing from women living with HIV	I think it would be a great idea to let them see what us, a woman that isn’t doing well, isn’t doing as well as the men definitely, overall, for them to see that there are women, that when we get it, most of us get real unhealthy real quickly.”
	Reminders and memory aids	“You could set your alarm on your phone, on your TV, in your car—they have everything that could literally like, “Oh, you need to take your medicine.””
	*Barriers*	
	Busyness	“I think there’s a much more important component, especially for woman. We usually think about everybody but ourselves, and, you know, it, and you get busy, you know, so even if you’re tryin’ to take, it’s hard for women, you know, maybe to take it on a regular, you know? Just ‘cause you get busy.”
	Low priority/ disinterest	“But there’s also a difference of if I’m sick and I take medicine, I’m willing to take medicine if I’m sick. If I’m not sick, I’m not as willing.”
	Perception of low risk for HIV	“I think one thing that you’re going to be fighting real hard is the misconception that the AIDS crisis is over. There’s a huge, huge, huge percentage of the younger population who’s sitting there going, “I don’t have to worry about AIDS. The AIDS thing’s over.”
	Unpleasant reminder of HIV or of risk behaviors	“Well, plus, as sexually speaking, it’s a reminder—if you’re taking this every day and you’re like, “I don’t want to get AIDS. I don’t want to get AIDS.”
	Poor access	“Well, that’s the biggest thing. If it’s not covered by insurance, not many people are gonna pay for it, and they’re not gonna go out of their way to pay for it and insurances aren’t gonna cover it…”
	HIV stigma	“No one is gonna have that in their purse. So first of all, people that knows me anyway, and if they know what it is, they’re gonna be like, “Oh, you got…Spread the rumors.”
	Medical mistrust	“It’s not good. If it really was good, like and that’s the truth, people would take it, like thousands… People would start takin’ it.”

### PrEP awareness

Across all focus groups none of the women had used PrEP before and there was limited knowledge about PrEP, with only a few individuals (n = 4) having heard about PrEP previously. Among those with some prior knowledge of PrEP, many conflated PrEP use among individuals without HIV and antiretroviral therapy (ART) among individuals living with HIV. For example, a White WLHIV in a serodiscordant relationship relayed her opinion based on her husband’s experience:

I don’t think enough long-term studies have been done with PrEP. Because I think, say you’re single…and you’re takin’ PrEP, and you’re sleepin’ around. Say 10 years from now you get married and you think, ‘I can stop takin’ PrEP.’ Do we know that you didn’t actually contract HIV during those 10 years and PrEP was just keeping it at bay? Because that’s what my husband’s regimen does.

Other women were suspicious about why they hadn’t previously heard about PrEP, suggesting that information about PrEP was being withheld from them for pernicious reasons. “So basically like all the doctors know about this? Why is this somethin’ that they haven’t presented to us when we go to the doctors appointments or things like that?” (Black woman without HIV). Despite this medical mistrust, women were generally curious to learn more about PrEP and posed clarifying questions (Table 1) around its efficacy, prescribed route and frequency of administration, safety (particularly during pregnancy) and prescription logistics (e.g., cost, insurance coverage, prescriber/pharmacy accessibility).

Participants also provided suggestions of ways to broaden awareness of PrEP among cisgender women (Table 1), including providing PrEP education in prisons, having PrEP support groups, using social media in conjunction with more traditional modes of advertising (e.g., television commercials, pamphlets, billboard advertisements) to expand the reach of PrEP messaging and providing information about PrEP in places women seek health and reproductive care (e.g., Planned Parenthood, gynecology offices). Because many felt that young women would be good candidates for PrEP, they thought it should be discussed in schools at all levels of education (Table 1). It was also suggested that women living with HIV would be vital sources of information about HIV and prevention and, in some ways, had a responsibility to share their stories and encourage new strategies for prevention. One White woman without HIV spoke to the WLHIV present in the same group, “We need to hear from you, people who are living with this…because I’m hearing that…yes, you’re living with it but, yes, you have limitations. Yes, your lives are very, very, different. Are you living—yes. But, you know, it’s not easy.” Furthermore, having now learned about PrEP, some participants expressed a sense of responsibility of needing to pass on the word about PrEP to other women, particularly their children—“I’m gonna tell my daughter about it. She’s 26. That’s my only child, so I’m gonna share it with her” (Black woman without HIV).

### PrEP uptake

Concomitant to discussing PrEP awareness and ways to develop it, participants voiced what they saw as potential drawbacks of using PrEP (Table 2). Specific concerns about PrEP included the experience of side effects, possibility of medication diversion, development of resistance, lack of protection against other STIs and the possibility of increased risky sexual behaviors among individuals on PrEP. Some expressed concern regarding gaps in information about PrEP, including a shortage of data on PrEP use during pregnancy. One Latina woman without HIV stated: “I think they’d want to see more studies with women and see…if there’s any… medical effects. You know, questions about like… could it affect if you get pregnant while you’re taking it?”.

Despite their concerns, most women cited a clear benefit to taking PrEP as an active positive step to protect themselves from HIV. “You know…it’s okay to… to protect yourself, put yourself first” (Black woman without HIV). Some viewed it as offering a wider repertoire of choices, beyond condoms or practicing abstinence, and providing peace of mind. Finally, participants thought PrEP use might facilitate intimacy in the context of discordant HIV statuses (Table 2).

Lastly, participants discussed the various groups of women that might benefit the most from taking PrEP. Mirroring their previous identification of individuals at risk for HIV, women took behavioral risk factors into consideration, stating that those engaging in sex work or IDU would be good PrEP candidates. Participants thought PrEP would be beneficial when a woman was disempowered and concerned about infidelity within her relationship. One Black woman without HIV said, “Even a poor mother on welfare is at home…takin’ care of her kids.…She’s poor but her man is out there doin’ things, so he can bring it back to her.” Participants also equated youth with promiscuity and thus suggested that young women with many sexual partners might benefit from PrEP. Additionally, participants acknowledged the demographics of the US epidemic, suggesting that women of color may particularly benefit from PrEP use. Furthermore, noting declining use of prevention methods among women who are no longer of reproductive age, they also suggested benefit of PrEP among older post-menopausal women (Table 2).

### Barriers and facilitators of PrEP initiation and adherence

Focus group participants also discussed factors that may impact initiation and adherence to PrEP. In particular, women described responsibilities associated with child rearing, expectations to attend to other people’s needs, stigma against HIV, poor access to PrEP and lack of physician support as potential barriers to PrEP use (Table 2). There was also a concern that taking daily PrEP might serve as an unwanted reminder about HIV/AIDS risk or might repeatedly highlight the conflict between their values and behaviors and result in cognitive dissonance: “So mentally, I think…that’s why youth are like, ‘I don’t want that,’ because it’s a constant reminder of what you’re doing is bad, or dangerous…” (White women without HIV).

On the other hand, women described ways to support taking PrEP, including using reminders and memory aids such as phone apps and pillboxes (Table 2). They also noted cognitive motivations like the desire to maintain their health and a sense of empowerment in directing their own sexual health would be supportive of PrEP use among women. They additionally noted that receiving social support from family, friends, and their medical providers would motivate their adherence (Table 2). An interesting finding across all the focus groups was a reiteration of the importance of promoting social support among women considering or taking PrEP (Table 2). In particular, women without HIV suggested that hearing from their peers on PrEP could encourage PrEP adherence: “Yeah, that would work because you are sittin’ with a whole bunch of people who are taking the same stuff you’re takin’, and they can say something that you probably never thought about yourself, and then… that might help you” (Black woman without HIV). They also suggested that hearing from women on ART could motivate taking PrEP, with some WLHIV remarking they would have taken PrEP had it been available to them: “…if I had been offered a pill 23 years ago, even though I wasn’t at risk, but I had the option of taking that pill to prevent myself from getting [HIV], I would probably have been one of the first in line, because this disease wreaks havoc on a person” (White WLHIV).

## Discussion

Risk for HIV acquisition among US cisgender women is not distributed equally, with women of color disproportionally bearing the burden of new infections [15]. However, when examined against the abundant literature among MSM, there is a relative lack of understanding in research of the needs and experiences of cisgender women with regards to using PrEP for HIV prophylaxis. Thus, identifying factors that influence PrEP uptake and adherence among racially and ethnically diverse cisgender women is central to curbing the US epidemic, improving PrEP utilization, and to designing effective interventions among this under-represented subpopulation of individuals at risk for HIV acquisition.

Focus groups, predominantly comprised of cisgender women negative for HIV infection, were carried out to understand PrEP awareness and to identify potential supports for PrEP uptake and adherence. In findings similar to those of previous qualitative studies among women, knowledge about PrEP was limited; however, women expressed high rates of interest in taking PrEP [9]. This continued mismatch between women’s awareness of and interest in PrEP may contribute to the continued low rates of PrEP utilization among cisgender women.

Women described anticipated benefits of using PrEP, including freedom from worry about HIV, the ability to engage in serodiscordant relationships, holding greater control over their sexual health, and broadened choices with regards to methods of HIV prevention. They also described a number of facilitators of PrEP initiation and adherence, including using reminder systems that are effective for other medications or behaviors and receiving social support from partners, family, friends, and medical providers. They also emphasized that connecting with other women taking PrEP and hearing from WLHIV would be support of their PrEP use.

However, discussion of the potential benefits of PrEP and of methods of improving using PrEP use were measured against concerns, such as potential increases in condomless sex, medication side effects, lack of protection against other STIs, and the absence of long-term follow-up studies on potential adverse events and toxicities of PrEP. Many of the barriers to PrEP uptake and adherence expressed by women echoed those reported in other studies among women and among MSM. For instance, women identified structural (e.g., limited accessibility of services, cost), cognitive (e.g., perceiving oneself to be at low HIV risk), and social barriers (e.g., lack of support from partners, concern about being misidentified as living with HIV) as impediments to initiating and adhering to PrEP [10, 16, 17]. Medical mistrust, specifically the concern women expressed over feeling PrEP was not offered or discussed by providers despite being available, also tempered enthusiasm for PrEP. Medical mistrust and HIV conspiracy beliefs have previously been reported as barriers to ART adherence, particularly among racial/ethnic minorities [9, 18]. As a result of a legacy of institutional racism within research and the medical system, there remains uncertainty, misinformation and mistrust about various aspects of HIV, particularly among Black and Latinx communities, which could ultimately affect widespread PrEP acceptability among the communities most vulnerable to HIV [19–22]. Thus, PrEP uptake and adherence may similarly be influenced by these factors [23].

Cisgender women were also uniquely concerned about whether PrEP could be taken during pregnancy and remarked on the perceived inadequacy of studies investigating the potential for reproductive harm. Although other qualitative work has reported expressed curiosity among women with regards to the safety of PrEP use during pregnancy, reproductive concerns were not previously prominently cited as barriers to PrEP use [24]. Other studies have highlighted reductions the ability to conceive healthy children when a male partner is living with HIV as a reproductive benefit of PrEP [11]. Lastly, underscoring the centrality of social and relational identities among women, participants also anticipated that pressure to fulfill social and occupational roles would interfere with their ability to prioritize their sexual health needs and hinder their ability to take PrEP consistently.

A number of recommendations emerged from the focus group discussions. In particular, women suggested methods of increasing PrEP awareness among members of the general public by using diverse modes of advertising (e.g., social media or online forums as well as television commercials and billboard advertisements). They also advised more targeted PrEP messaging, tied to their perceptions of who is at greatest risk of acquiring HIV, either by their behavioral characteristics (e.g., multiple sex partners, sex workers, IDU), belongingness to vulnerable demographic strata (e.g., youth and young women in particular, ethnic/racial minorities) or dyadic characteristics (e.g., women in serodiscordant relationships or those in relationships with doubt about their sexual partners’ faithfulness). Women suggested increased advertising about PrEP in spaces frequented by those at risk for HIV such as schools and prisons as well as places where women seek reproductive care including Planned Parenthood and at gynecologists’ offices. Similar to suggestions to support women who are on PrEP, participants advocated for hearing from other women, particularly those living with HIV, to further foster interest in taking PrEP. Thus, increasing PrEP messaging and varying its modes of delivery, while also emphasizing the benefits of using PrEP articulated by women, may drive PrEP uptake among cisgender women.

An interesting finding from the first focus group with women of mixed serostatus was that WLHIV would have been interested in taking a prevention pill had one existed when they were younger. This finding is in contrast to results from a previously-published qualitative study which reported that WLHIV were reluctant to recommend PrEP to women without HIV, citing reasons including access, cost, potential side effects and the ability to simply use condoms instead [25]. These disparate results could reflect the diversity in geographic settings or participants’ differing experiences with HIV medications; however, they could also be related to the structure of the focus groups, with our study having WLHIV and women without HIV infection together as opposed to segregated by serostatus, as in the previous study.

In summary, this study corroborates and extends prior work examining PrEP attitudes and knowledge and also identifies strategies to potentially improve PrEP uptake and adherence among women. Our findings suggest a need to emphasize that cisgender women can be at increased risk for HIV, as they remain relatively unaware of PrEP, and suggests ways of altering current messaging about HIV prevention for cisgender women. Concerns raised by women in our focus groups suggest that, to engage racially and ethnically diverse female audiences, PrEP information will need to directly address reproductive safety, include representation by women other women can relate and attend to, and be widely disseminated in places where at-risk women will notice. Efforts to provide PrEP education must also address HIV knowledge, medical mistrust, and HIV stigma. The varied strategies suggested by women to improve PrEP awareness may be important for disseminating PrEP knowledge, given the difficulty in having cisgender women self-identify as at-risk and of having health care providers identify cisgender women who are at increased risk for HIV acquisition.

Our study findings should be considered considering its limitations. First, our participant sample was small, limiting the generalizability of our findings and potentially limiting the ability to reach saturation of themes. Second, as we only utilized focus groups, we may not have been able to achieve the same degree of insight as with a combination of in-depth key informant interviews and focus groups. However, these focus groups were embedded within the context of a larger demonstration study, with results forthcoming (clinicaltrials.gov NCT02584140). Future work should consider utilizing a larger sample and multimethod qualitative approaches. Finally, we recognize that these focus group were held 5 years ago. Even though PrEP is rapidly evolving for many populations, progress for cisgender women in the US has unfortunately been extremely limited. Therefore, these data are still relevant and supplement the current literature as focus groups add to the richness of the existing quantitative data.

## Conclusion

Despite these limitations, this formative assessment was able to obtain novel perspectives that may contribute to knowledge on PrEP among racially and ethnically diverse women. In particular, despite limited PrEP knowledge and acknowledgement of concerns and potential barriers to taking PrEP, cisgender women participating in focus groups highlighted the importance of PrEP for supporting their sexual health and identified ways to facilitate PrEP uptake and adherence. Examining the opinions of cisgender women is essential for the development of supportive interventions and programs to support their PrEP utilization.

## Supplementary Information

**Additional file 1**. Focus Group Guide.

## Data Availability

The data that support the findings of this study are available through MAXQDA 2017 (VERBI Software). Restrictions apply to the availability of these data, which were used under license during this study. Data are available upon request from the corresponding author.

## Abbreviations

ART: Antiretroviral therapy
IDU: Injection drug use
MSM: Men who have sex with men
PrEP: Pre-exposure prophylaxis
STI: Sexually transmitted infections
TDF/FTC: Tenofovir disoproxil fumarate with emtricitabine
TGW: Transgender women
WLHIV: Women living HIV
