# Supplementary material for: “PrEP”ing for a PrEP demonstration project: understanding PrEP knowledge and attitudes among cisgender women
Source: BMC Womens Health. 2021 May 25;21:220. doi: 10.1186/s12905-021-01348-8 (PMC8146684; doi:10.1186/s12905-021-01348-8)
Supplement: Supplementary file 1 — Additional file 1. Focus Group Guide. [file 12905_2021_1348_MOESM1_ESM.docx]

**HIV Prevention Discussion Group on Adherence Support for Pre-exposure Prophylaxis (PrEP) Study among Women**

Version 1.0 dated 10/02/2015

RESEARCH GOALS

- Explore critical benefits and barriers, as perceived by women, for using PrEP as an HIV prevention strategy
- Explore perceptions of needs and preferences for adherence support

1. Introduction

Purpose

Hi. Thanks for joining our group. My name is _____________ and I’ll be leading this discussion group today. Have any of you talked in a group like this before?

This is called a “focus group.” It’s a way for us to hear what you have to say when we conduct research that is supposed to help women like you. We’ll talk for about an hour and 30 minutes.

We’re going to talk about what it’s like to be a woman in CITY, especially when it comes to HIV prevention. We want to hear about what you do to prevent yourself from getting HIV and to protect your sexual health. Also, we want to know your opinions about an HIV prevention method that involves taking a pill every day to help prevent HIV. We’ll talk more about how we plan to use this information later on.

Right now, I want to let you know a few things about what we’re doing:

Disclosure

- Audio taping
- Reporting
- Observers helping to listen/take notes
- Participant information sheets

Procedures

- This is a safe place for sharing. All personal information that is discussed in this room is to remain between the participants here. Please do not share this personal information with anyone outside of this discussion group. You are welcome to talk about any factual information that you learn about HIV prevention that may be of use to your friends.
  - Do you agree to not share any of the personal information discussed here with anyone outside of this discussion group?
    - Each participant gives his verbal agreement.
- No right or wrong answers. We want to hear your personal opinions. If you have any questions about HIV preventions methods, the principal investigator __________ is here to help answer them.
- Be honest. We want to know what you really think.
- We want to hear from everyone, so please don’t be shy.
- No need to raise your hand to speak.
- One person talks at a time.
  - Please silence your cell phone and refrain from using it during the group.
- No official breaks. If you need to get up to use the restroom, please do so as quietly as possible. It is located ________________________.
- You may choose to leave the discussion group at any time, and this will not impact your relationship with UCLA in any way. Also, you may choose to not answer any question that you do not want to answer without any penalty.
- At the end of the discussion group, you will be compensated for your participation.

Information Sheet

- Please take a few minutes to review the information sheet. This is the same information sheet emailed to you prior to today’s discussion group. This is your copy to take home with you when you leave tonight.
- Any questions?

1. HIV Prevention Information

Ok, I want to start by asking you some questions about ways that you protect yourselves from HIV. First, I want to know:

- - 1. Tell me what you know about strategies to prevent HIV.
       1. Probe: What do you know about behavioral strategies to prevention HIV? By behavioral, I mean behaviors you may change to limit your risk of getting HIV, such as using condoms, avoiding sex, or speaking with your partners about HIV. (e.g., condoms, abstinence, partner testing and disclosure, etc.)
       2. Probe: What do you know about biomedical HIV prevention? By “biomedical prevention” I mean pills that are taken orally or gels that can are inserted into the vagina that help protect against HIV. (e.g., vaccines, pills, microbicides)
          1. Probe: Have you used any of these strategies? Which ones?
          2. Probe: How did you obtain them? (e.g., clinical trial, PCP, etc.)
       3. Probe: Tell me about what’s important to you when choosing an HIV protection strategy. How do you choose which strategy to use?

1. Barriers / facilitators of PrEP / daily oral HIV prevention and adherence support

Some of you mentioned that you have heard of “PrEP”, or pre-exposure prophylaxis for HIV, and some of you may have even used it before. To get everyone on the same page, for the purposes of our discussion, we will be talking about oral PrEP. That is, the use of an antiretroviral medication, to *prevent* HIV. Antiretroviral medications are the class of medications used to treat HIV. Research has shown that if HIV negative individuals take an antiretroviral medication called Truvada on a daily basis, it reduces the risk of getting HIV dramatically.

We are going to be conducting a study looking at how acceptable and effective PrEP is for women living in California, and we would like to ask you some questions about things that could get in the way of you using PrEP and things that may help you use PrEP.

There are a few things about PrEP use that I would like to share with you that may impact your answers to my questions:

- PrEP is currently prescribed as a once a day pill.
- Adherence, or the ability to take PrEP as it is prescribed, has a big impact on its effectiveness. In other words, the people that take it as close to once a day as possible tend to do better with respect to HIV prevention.
- PrEP does not protect against other sexually transmitted infections, or STIs/STDs, nor does PrEP prevent pregnancy.
- PrEP generally has very few side effects and is considered very safe. However, PrEP users need to have a physical and be tested for HIV before starting PrEP.
- PrEP users also need to see their health care providers regularly for monitoring.
- PrEP is covered by some insurances, but not all.
- What questions do you have?

1. How would you feel about taking PrEP?
2. I would now like to hear about things that might get in the way of you using PrEP as an HIV prevention strategy. It may be helpful to think about your experiences using other once a day medications such as birth control or antibiotics, but, it is also important to think about the ways that preventing HIV is different from taking birth control or antibiotics.
   1. Probe: what about (ask about each of the following):
      1. Burden of daily dosing
      2. Stigma / privacy concerns
      3. Messages from community / partner(s) / friends
      4. Not being able to engage consistently with health care providers (assess reasons, e.g., childcare and/or work demands)
      5. Substance use / abuse (assess which ones)
      6. Mental health (e.g., depression)
      7. Forgetting (assess when most likely)
      8. Changes in sexual activity
3. What are some things that might help you take PrEP?
   1. Probe: What things could help you take PrEP as prescribed, or once per day?
      1. Probe: Reminder aids (e.g., cell phone reminders, texts, etc.)
   2. Probe: What role, if any, would your partner(s) play in your taking PrEP?
   3. Probe: What has helped you take other once a day medications in the past?
4. Because adherence is so important to the effectiveness of PrEP, in our study we would like to offer participants adherence support. By adherence support, we mean that we would offer our participants specific opportunities to speak with a study staff member about ways to take PrEP effectively, and to talk about things that may make taking PrEP consistently difficult.
   1. Probe: Tell me what you think about this.
   2. Probe: What should this support look like? What kind of support or counseling would be helpful?
   3. Probe: What would make this support be effective?
   4. Probe: Who should deliver this support?
   5. Probe: How often should support be provided?
   6. Probe: How long should these conversations last?
   7. Probe: Where / how should this support occur (e.g., in person, via phone, etc.)
5. Conclusions
   1. Does anyone have anything else they would like to add to our discussion group today?

Thank you so much for your help. Your feedback is really useful to us. Before you leave tonight, we would love for you to fill out this brief survey with your basic information. None of this will be used to identify you.

Also, if you would like more information about HIV prevention studies at our clinic or other sites throughout CITY, or to be notified about similar discussion groups in the future, please fill out the separate contact sheet with your first name, email address and/or phone number. Thank you again!
